# Innovative Family-Based Genetically Informed Series of Analyses of Whole-Exome Data Supports Likely Inheritance for Grammar in Children with Specific Language Impairment

**DOI:** 10.3390/children10071119

**Published:** 2023-06-28

**Authors:** Erin M. Andres, Kathleen Kelsey Earnest, Hao Xuan, Cuncong Zhong, Mabel L. Rice, Muhammad Hashim Raza

**Affiliations:** 1 Thompson Center for Autism and Neurodevelopment, University of Missouri, Columbia, MO 65201, USA; eandres@health.missouri.edu; 2 Language Acquisition Studies Lab, University of Kansas, Lawrence, KS 66045, USA; 3 Department of Electrical Engineering and Computer Science, University of Kansas, Lawrence, KS 66045, USA; 4 Child Language Doctoral Program, University of Kansas, Lawrence, KS 66045, USA

**Keywords:** whole-exome sequencing, language phenotypes, specific language impairment, family-based, pedigree, grammar impairment

## Abstract

Individuals with specific language impairment (SLI) struggle with language acquisition despite average non-verbal intelligence and otherwise typical development. One SLI account focuses on grammar acquisition delay. The current study aimed to detect novel rare genetic variants associated with performance on a grammar assessment, the Test of Early Grammatical Impairment (TEGI), in English-speaking children. The TEGI was selected due to its sensitivity and specificity, consistently high heritability estimates, and its absence from all but one molecular genetic study. We performed whole exome sequencing (WES) in eight families with SLI (*n* = 74 total) and follow-up Sanger sequencing in additional unrelated probands (*n* = 146). We prioritized rare exonic variants shared by individuals with low TEGI performance (*n* = 34) from at least two families under two filtering workflows: (1) novel and (2) previously reported candidate genes. Candidate variants were observed on six new genes (*PDHA2*, *PCDHB3*, *FURIN*, *NOL6*, *IQGAP3*, and *BAHCC1*), and two genes previously reported for overall language ability (*GLI3* and *FLNB*). We specifically suggest *PCDHB3*, a protocadherin gene, and *NOL6* are critical for ribosome synthesis, as they are important targets of SLI investigation. The proposed SLI candidate genes associated with TEGI performance emphasize the utility of precise phenotyping and family-based genetic study.

## 1. Introduction

Is human language inherited? Familial aggregation and behavioral genetic studies have consistently suggested that genes have a greater influence on language expression than the environment [1,2,3,4,5,6]. The current study adds supportive evidence to the claims of the innateness of language and its specificity to humans, specifically adding to our understanding of the genetic basis of specific language impairment (SLI). SLI is characterized by a delay in language acquisition and a persistent language deficit in the absence of hearing loss and other neurological or developmental disorders [7]. The estimated prevalence of SLI is 7–10% in English-speaking populations [8,9]. SLI remains a valid phenotype in the scientific literature, but we also note a recently updated term, developmental language disorder (DLD), which includes those who do not meet the specific criteria for SLI, as well as those who do meet the diagnostic criteria for SLI [10,11]. The advantage of the SLI criteria is a greater measurement precision for group status. Categorical labels for children with language impairments can be viewed as clinical labels for eligibility for services in contrast to group labels for scientific studies such as the one reported here. Further, the pathways differ for clinical services across countries. In some countries, they are nested within school special education services (e.g., the United States of America (USA)), whereas, in other countries, they are nested within public health/medical services (e.g., the United Kingdom (UK)). In the USA, the term ‘SLI’ arose as a scientific label to differentiate children with multiple developmental disorders from children whose language disorders are their single developmental disorder [12]. ‘Developmental Language Disorders’ could be applied to children with co-existing clinical neurological disorders [13].

Discussions surrounding standardizing the diagnostic criteria for SLI/DLD have tended toward increasing the language and intelligence standard score cut-offs and many have discussed how variable diagnostic criteria have impacted our continued questions about the genetics of language acquisition [14]. In contrast, the current study defines the SLI phenotype according to performance on the Test of Early Grammatical Impairment (TEGI), a sensitive and specific assessment of tense and agreement marking abilities in individuals speaking mainstream American English [15]. A deficit in tense and agreement marking is a known clinical behavioral marker of SLI [16,17]. The emphasis on the grammar deficit also supports our interest in the genetic influences specifically on language acquisition, given that the participants represented in the current study show a deficit in language despite average or above average non-verbal intelligence [16,18,19]. There is continued interest in strengthening our interdisciplinary approaches to speech and language impairments, especially in thinking about how speech–language pathologists can expand and use their knowledge of behavioral genetics in practice [20]. Additionally, there has been a push for utilizing larger cohorts of unrelated individuals and existing medical records for the genetic investigation of language traits [21,22,23]. Significantly, our study relies foremost on the sensitivity and specificity of the TEGI and the genetic relatedness of the participants and the power it may provide to the genetic investigation of SLI.

The TEGI is regarded as one of the most psychometrically sound instruments in terms of sensitivity and specificity in the assessment of children with SLI (after age three) [15,24,25,26]. The TEGI was developed through a longitudinal study, and the evidence supporting the specificity of the test is rooted in the linguistic theory of grammar acquisition [15,17,27]. Specifically, Wexler’s (1994) theory of optional infinitive (OI), which is based on the assumption of Universal Grammar (UG)-constrained Maturation (UGCM) [27], contributed to the development of the TEGI and the theory of a clinical linguistic marker of SLI [17]. UGCM assumes children have an innate capacity for adult grammar, which matures, but during that maturation, it generally does not allow the child to produce utterances that go against the UG [27]. The OI theory focuses on the optionality that children appear to have in their grammar, causing them to produce infinitive verb forms (e.g., “She teach”), when morphological endings are obligatory [27]. The absence of inappropriate inflection supports the assumption of UGCM [27]. Children with SLI show growth trajectories of multiple language abilities that run parallel to their typically developing peers (same slope of development) about two years delayed [16,17]. The delay period was observed to extend the time that children with SLI optionally use tense, motivating the theory of Extended OI (EOI) and EOI as a clinical marker of SLI [16,17,28]. A recent report of individuals with SLI from the current study and additional individuals from the larger longitudinal study provides evidence that the difficulty in finiteness marking observed in childhood extends through age 18, as measured by performance on tasks with more grammatically challenging linguistic structures [29]. Crucially relevant to the current study, receptive and expressive grammar phenotypes revealed significant heritability estimates (up to 0.92) in two twin cohorts at time points ranging from 2 to 16 years [3,4,5,30]. Despite significant heritability estimates, the TEGI phenotype was used only once across previous molecular genetic investigations of SLI [31]. The current study aims to prioritize rare genetic variants from whole-exome sequencing (WES) data that segregate with the TEGI phenotype. 

Two epidemiological twin cohorts tested for grammar impairment (i.e., neither ascertained based on SLI status) revealed that the lowest performing groups yielded the highest heritability estimates [3,4,5]. These results suggest that genetic factors may explain more of the variance in grammar abilities among individuals with language impairment (LI) than in those without LI and that grammar impairment may be the most valid clinical marker in need of study at the molecular genetic level [4,5].

Family-based linkage studies provided several gene targets and multiple genes have been suggested for SLI through WES [18,19,32,33]. Family-based studies, in conjunction with next-generation sequencing (NGS), have the potential to provide promising gene targets to explain the biological basis of language acquisition. Three studies of SLI utilizing WES output are of note. First, WES of select individuals (*n* = 5) from a founder population of Robinson Crusoe Island (*N* = 117) resulted in a strong candidate, *NFXL1* [33], which expresses in the cerebellum, a region previously implicated in language development [34]. Second, another study used multiple variant filtering criteria to identify variants of interest from WES output from select SLI Consortium probands (*n* = 43) [32]. One variant prioritization approach targeted variants within candidate genes previously suggested for SLI, as recommended by the guidelines put forth for evaluating the causality of sequencing variants [32,35]. The other streams of variant filtering prioritization identified rare stop-gain variants, with bioinformatic in silico scores predicted to be deleterious, and sought compound heterozygotes and cases of what the authors called “multiple-hits” [32]. Ultimately, the WES findings led to the hypothesis that transmission of a complex disorder, like SLI, is likely to be explained by a combination of genetic variants (rare and common), including those on previously identified genes [32]. If related individuals were included at the whole-exome level, co-segregation analysis could have been used to prioritize variants. Third, we recently used a similar approach in the study of family 5886 (reported as family 489); we used three workflows to prioritize rare variants of interest and observed a co-segregating protein-coding rare variant in *BUD13*, which was not previously reported for SLI or related phenotypes. Targeted sequencing of this gene in unrelated SLI probands from the same population revealed more *BUD13* variants in additional probands with statistical significance [19]. Overall, these findings support the value of WES investigation, especially utilizing related individuals who are both affected and unaffected, to identify novel SLI candidate genes. 

**Figure 1:**
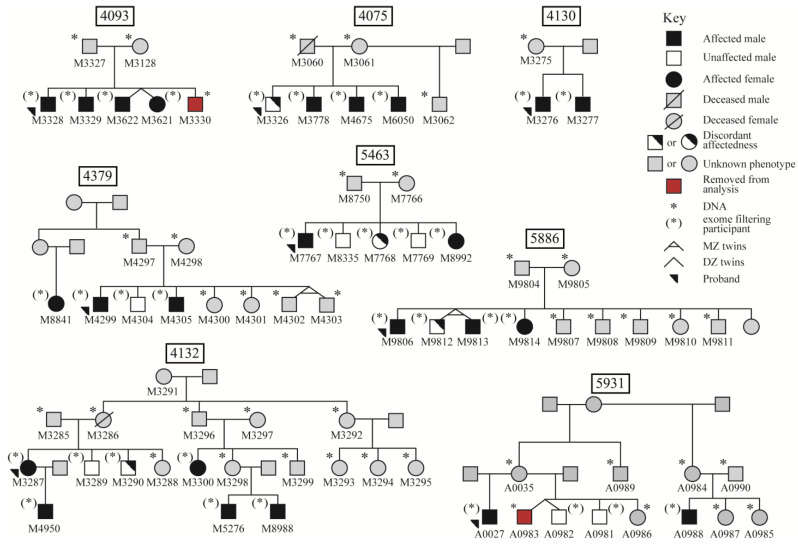
Eight Families included in WES (*n* = 74) with Categorical Affectedness Status for the TEGI in a Subset of the Family Members (*n* = 34). Note. Discordant affectedness refers to performance in the unaffected range on the screener or composite probes, but not both. Family 4132 Branch 1 (proband branch) includes descendants of M3286 and M3285, Family 4132 Branch 2 includes descendants of M3296 and M3297, and Family 4132 Branch 3 includes descendants of M3292. Family 5931 Branch 1 (proband branch) includes descendants of A0035, and Family 5931 Branch 2 includes descendants of A0984 and A0990. One proband (M3287), in family 4132, had only a TEGI screener score available. Another proband (M3326) performed in the unaffected range on the TEGI screener probes, but their composite performance was in the affected range.

**Figure 2:**
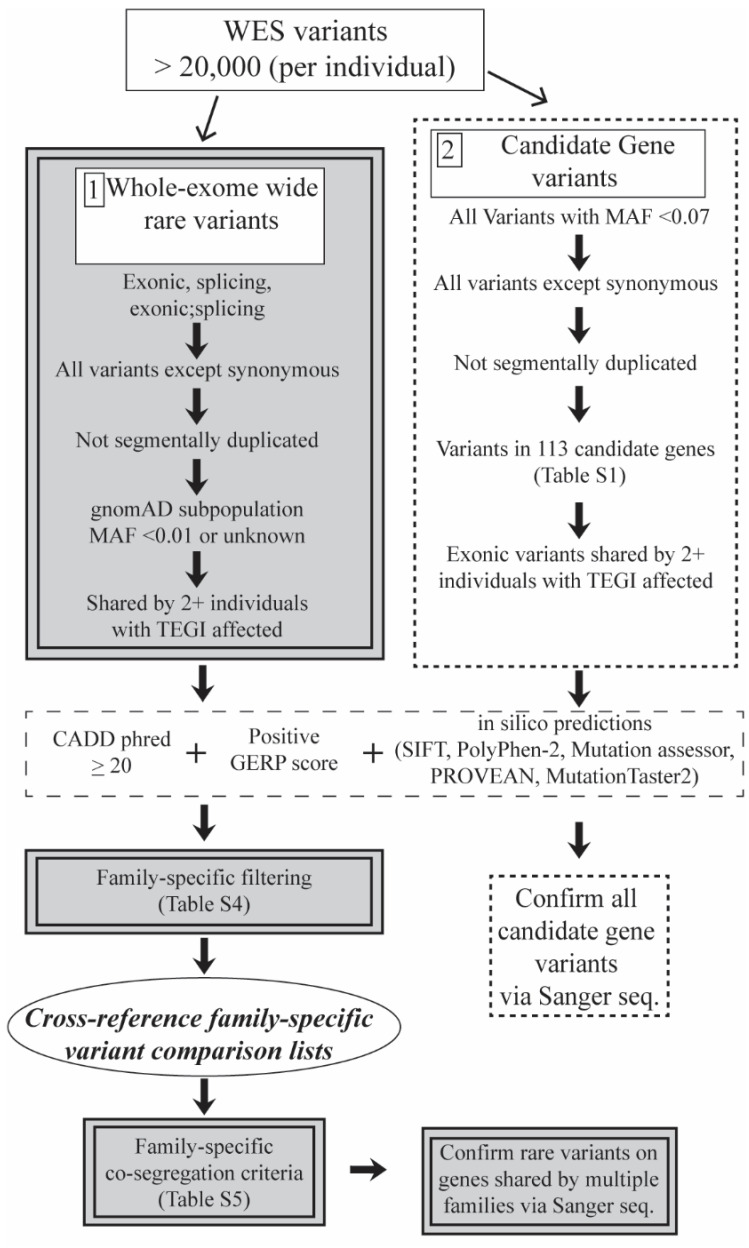
Variant Prioritization Workflow. MAF = minor allele frequency; familywise ‘co-segregating variant lists’ presented in Table S11a–h.

In the current study, we utilized WES output from select individuals who have completed the TEGI, from eight informative families from the University of Kansas (KU) SLI cohort (Figure 1, *n* = 74). Note genetic findings from many of these families have been reported previously [18,19,31,36]. We focused our genetic analysis on (Figure 2): (1) novel candidate genes and (2) variants in 113 previously reported candidate genes implicated in SLI and related phenotypes (Table S1) in the family members who have completed the TEGI (Table S2, *n* = 34). We hypothesized that the sensitivity and specificity of the TEGI and the consistent reports of high heritability would support the precise detection of rare genetic variants associated with SLI. We prioritized variants shared by at least two families and predicted the identified variants may also be observed in the larger KU SLI cohort, who have completed the TEGI (Table S3, *n* = 146).

## 2. Materials and Methods

The institutional review board (IRB #8223) at the University of Kansas approved this study for behavioral data collection on 25 January 1993, and it has been annually reviewed and approved since that time. Relevant genetics amendments included: (1) Collection of DNA via blood draw and cheek cell samples, approved on 19 January 1999; (2) Collection of DNA via saliva samples, approved on 31 January 2006; (3) consent form update to include the National Institutes of Health Certificate of Confidentiality, approved on 3 January 2018. Participants provide their signatures for informed consent to all amended genetics protocols. All methods were performed in accordance with the relevant guidelines and regulations of the Declaration of Helsinki and University of Kansas Human Research Protection Program. All participants provided appropriate informed consent. 

Participants in the current study were part of a larger ongoing longitudinal study of probands with SLI and their family members. Details concerning all assessments administered as part of the larger study are described in an earlier publication by Rice, Smith, and colleagues [31]. The term ‘proband’ refers to the individual originally targeted for the study. The proband entrance criteria for the study include (i) average or above average performance on a standardized non-verbal intelligence (NV-IQ) measure (standard score > 85), (ii) typical hearing, (iii) no history of neurological disorders or autism diagnosis, and (iv) intelligible speech/articulation [18,19,31]. All participants are monolingual speakers of General American English [18,19]. 

Individuals from eight families in the larger study *(n =* 74) were included in the current study. A subset of the individuals (*n* = 36) have completed the TEGI (age-referenced for children ages 3 to 8; 11 years), the phenotype of interest, at least once (Figure 1) [15]. Two siblings (both males) were excluded from the subset due to potentially confounding patterns of lowered NV-IQ (Figure 1). The remaining individuals (Table S2; *n* = 34; 27 males and 7 females; referred to as TEGI-WES group) were the focus of the WES variant filtering (Figure 2). All eight probands (7 males and 1 female) are affected on the TEGI based on their elicited grammar composite or screener score, six of whom are affected according to both scores (Figure 1). 

The current study assigned affection status categorically based on the participants’ lowest score across time points, consistent with how previous research established affectedness [18,19]. The TEGI probes tense marking and finiteness relative to mastery in adult grammar [15]. The screener and composite probes require elicitation. A phonological probe prior to the other probes ensures that the child can produce the required sounds; crucial because the marking is required in the final position of the word [15]. The screener score is the average of the third-person singular and past tense probes, while the elicited grammar composite score also includes the ‘be’ and ‘do’ probe scores [15].

The total number of additional probands available for participation in this study was 157 (provided DNA and completed the TEGI). Eleven children who may have shown potentially confounding patterns of lowered NV-IQ were excluded from our analysis, resulting in a total of 146 probands. The proband entrance criteria do not require the proband to score in the affected range on the TEGI. Therefore, 21 probands included did not show low performance on the TEGI (Table S3). The TEGI-WES group (*n* = 34) and the additional probands *(n* = 146) all completed an age-appropriate standardized omnibus language measure and a receptive vocabulary measure (Tables S2 and S3).

Participants provided saliva samples/buccal swabs using the Oragene-Discover OGR-500 or OGR-575 Kits (DNA Genotek, Oragene). DNA was purified according to the manufacturer’s instructions. WES and bioinformatic analyses were performed in eight families (*n* = 74) over two-time points. The first round of WES was performed in select individuals from six of the eight families (*n* = 29), using the Illumina Nextera Rapid Capture Enrichment kit (expanded; includes untranslated genomic regions [UTR]). The remaining individuals from the six families and all individuals from two additional families (*n* = 45) were included in the second round of WES using the Illumina NovaSeq6000 (UTRs were not included). The sequencing data were mapped to the human reference genome (hg38), and variants were called as described in an earlier publication [19].

The exonic variant filtering relied on categorical affectedness status based on TEGI performance (Table S2). Figure 2 shows the two complementary variant filtering prioritization approaches: (1) whole-exome wide (novel candidate genes) and (2) targeted prioritization (candidate genes previously reported for SLI and related phenotypes). Both approaches employ criteria to prioritize variants shared by multiple affected individuals within a single family. We applied the following common *a priori* filtering criteria in workflow 1 and 2: (i) classified as ‘exonic’, ‘splicing’, or ‘exonic;splicing’; (ii) not classified as synonymous; (iii) not located within a segmentally duplicated region; (iv) a Combined Annotation Dependent Depletion (CADD) Phred score ≥ 20; (v) a positive Genomic Evolutionary Rate Profiling (GERP) score; (vi) shared by at least two family members affected on the TEGI; (vii) multiple damaging scores according to five in silico programs, including SIFT (Sorting Intolerant from Tolerant), PolyPhen-2 (Polymorphism Phenotyping v2), Mutation Assessor, PROVEAN (Protein Variation Effect Analyzer), and MutationTaster2 [37,38,39,40,41,42,43,44]. Articles commonly assess and present multiple in silico prediction scores to provide context for the significance of the identified variants [19,36,44,45,46,47]. Finally, we applied family-specific criteria (detailed in Tables S4 and S5). All cross-referencing steps were completed in R using the ‘dpylr’ and ‘tidyr’ packages [48,49,50].

The first variant filtering workflow prioritized rare and novel variants whole-exome wide. Rare variants were defined as those with a minor allele frequency (MAF) ≤ 0.01 in the subpopulation appropriate for the family within the Genome Aggregation Database (gnomAD). Variants with unknown MAF and a predicted deleterious effect were defined as novel variants. Family 5931 is of African American descent, while the other families are of European descent. 

We cross-referenced ‘family-specific variant comparison lists’ to identify genes shared across the eight families (Figure 2; Table S4). Family-specific co-segregation criteria (Figure 2; Table S5) were applied (‘co-segregating variant lists’) to further reduce the prioritized list. In total, four individuals (across two families) with low performance on the TEGI were required to carry variant(s) on the genes on the final list of prioritized variants. 

The second variant filtering workflow independently prioritized variants in 113 candidate genes compiled from reviews and candidate gene investigations, as recommended by MacArthur and colleagues (Table S1; used in our previous WES investigation of family 5886) [32,35,51,52]. If the candidate gene was also listed in a more recent review (Mountford et al., 2022), the reference is noted in Table S1 [14]. Within the targeted filtering workflow, we prioritized variants using a less stringent MAF of <0.07 (Figure 2). Using a less stringent MAF applied to the updated public databases allows the filtering workflow to pick up variants in the previously suggested genes, such that confirming and disconfirming evidence for the previous candidates can be added to the literature.

After the variants with a MAF > 0.07, variants causing a synonymous change or variants located in a segmentally duplicated region were removed, the remaining variants were cross-referenced with the list of 113 candidate genes (Table S1) [48,49]. All variants on previously reported candidate genes that were shared by two individuals who showed low performance on the TEGI and met all other *a priori* filtering criteria were prioritized for confirmation via Sanger sequencing. Prioritized variants in the candidate genes were not required to be shared by two families, given that they share the gene with a previous report.

Oligos were designed using Primer 3 to amplify and confirm the prioritized variants (Table S6). Then, we analyzed the Sanger sequencing data in SeqMan Pro within the DNAStar suite. 

Select confirmed variants were Sanger sequenced in the additional probands (*n* = 146). Finally, variants were classified as benign, likely pathogenic based on if they met a combination of *a priori* criteria. The TEGI was completed by a subset of each family, which limited co-segregation analysis, so we added the likely pathogenic category based solely on the predicted pathogenicity.

## 3. Results

We prioritized variants in 36 genes by applying filtering criteria to the WES data in eight families (workflow 1 = 23 (Tables S7–S10 and S11a–h); workflow 2 = 13 (Tables S12 and S13)). Following confirmation, 12 variants in nine genes co-segregated in their respective families, prompting follow-up sequencing in the additional probands (*n* = 146; Table 1). We observed multiple unrelated probands carrying variants in six genes not previously reported for language impairment (*PDHA2*, *PCDHB3*, *FURIN*, *NOL6*, *IQGAP3*, and *BAHCC1*). We also observed variants in two genes previously suggested for SLI and related phenotypes (*GLI3* and *FLNB*). 

### 3.1. Variant Prioritization Workflow 1: Whole-Exome Wide Rare Variants

The eight families started with a range of 12,000 to just over 47,000 exonic variants (Table S7). When only the variants shared by two individuals with low performance on the TEGI were kept, the variants were reduced to under 700 for all families (Table S7). Then, family-specific filtering criteria (Table S4) reduced the ‘family-specific variant comparison lists’ to a range of 18 to 208 variants, and the unique genes were cross-referenced to reveal 55 shared genes (Table S7). Of the 55 shared genes, 8 genes were excluded for various reasons (described in Table S7). Family-specific co-segregation criteria (Table S5) further reduced the ‘co-segregation variant lists’ to a range of 6 to 37 variants (Table S7). The ‘co-segregating variant lists’ (Table S11a–h) were cross-referenced familywise with the 47 shared genes of interest (Table S8) and only 23 genes containing a variant in at least one family’s ‘co-segregating variant lists’ were further investigated (Tables S9 and S10). Seven genes were excluded according to the reported protein expression (Table S9). Variants on nine genes were either observed in all family members, were not confirmed, or the primers could not be optimized (Table S9). Variants on the remaining nine genes were confirmed through Sanger sequencing (Tables S9 and S10). Co-segregation of variants on six of these genes (*PDHA2*, *PCDHB3*, *FURIN*, *NOL6*, *IQGAP3*, and *BAHCC1*) with the TEGI was confirmed (Tables S9 and S10 and Table 1).

### 3.2. Variant Prioritization Workflow 2: Candidate Gene Variants 

A filtered list of variants (5000 to 29,000) was cross-referenced familywise with the established list of 113 candidate genes suggested for SLI and related phenotypes (Tables S1 and S12). No additional family-specific criteria were applied to filter the variants and in total, 14 variants in 13 previously reported genes were prioritized under filtering workflow 2 (Table S12). Variants on 12 of the candidate genes were prioritized for confirmation in family members via Sanger sequencing (variant shared by family 4132 and 5886 on *PTEN* was excluded; Table S13). 

In sum, all 13 variants on 12 candidate genes were confirmed in their respective families via Sanger sequencing (Tables S12 and S13). Co-segregation analysis with the TEGI was confirmed for variants in three genes (*GLI3*, *FLNB*, and *KMT2D*; Table 1, Tables S12 and S13).

### 3.3. Significance of Identified Variants in Additional Unrelated Probands

We Sanger sequenced 12 variants in nine genes (workflow 1 = nine variants in six genes and workflow 2 = three variants in three genes) in the additional probands (*n* = 146; Table 1). We observed variants in four genes from workflow 1 (*PDHA2*, *PCDHB3*, *NOL6*, and *IQGAP3*) in six additional probands (Table 1). A variant (rs35364414) in the candidate gene, *GLI3* from filtering workflow 2 was confirmed in 10 additional probands; four of these probands were not affected according to their TEGI composite or screener performance (Table 1). Another variant in *FLNB* was observed in two additional probands (Table 1). We performed a Fisher’s exact test comparing the variant counts in the total number of discovery probands (*n* = 8) and additional probands sequenced (*n* = 146) to the variant counts reported in gnomAD for the non-Finnish European subpopulation. The observed variants on *GLI3*, *FLNB*, *PDHA2*, *PCDHB3*, *FURIN*, and *IQGAP3* in unrelated probands were not significantly different from the gnomAD reports (*p* > 0.05; Table 1). The variant observed on *NOL6* in family 4130 and an additional proband (rs114465306) has not been observed in the non-Finnish European subpopulation (Table 1).

In total, 17 additional probands carried a prioritized variant on a previously reported gene or a newly prioritized gene (Table 1). One additional proband carried two variants (on *GLI3* and *PDHA2*); the proband was affected according to their TEGI composite and receptive vocabulary performance but performed well on an omnibus measure and the TEGI screener. Four of the 17 probands were unaffected on both the TEGI composite and screener and all carried the common (gnomAD non-Finnish European subpopulation MAF = 0.0502) variant on *GLI3* (rs35364414; Table 1). Though the sample size of additional probands is small (*n* = 146) and the majority are affected on all four phenotypes of interest (*n* = 77; Table S7), the probands unaffected on the TEGI composite and screener were more likely to carry the common variant than a rare variant (Table 1).

## 4. Discussion

This family-based molecular genetic study of SLI defined the phenotype based on low performance on the Test of Early Grammatical Impairment (TEGI). The TEGI measures a particular part of the English grammar and has high specificity and sensitivity for distinguishing between children with and without SLI between the ages of 3 to 8; 11 years [15,24,25]. The TEGI phenotype allowed for the prioritization of rare variants on multiple genes not previously suggested for SLI and precise detection of variants involved in SLI as defined by a grammar phenotype. The identification of multiple rare and common variants within single families supports the hypothesis that a single variant (or even only rare variants) may not be able to explain the genetic basis of SLI on its own. Further, the current study underlines a continued role for family-based genetic study in the pursuit of genes involved in disordered language acquisition.

Select individuals in the current study were included in four previous genetic investigations [18,19,31,36]. The first utilized the TEGI phenotype; Rice, Smith, and Gayán (2009) performed targeted linkage and association analyses of regions previously associated with a reading disorder (RD) in a large portion of the KU SLI cohort (*N* = 322). The relatedness was not explicitly accounted for in the analyses. There was significant linkage at chr6p22 and marginally significant linkage at a portion of the targeted chr3p12-q13 region to the TEGI composite phenotype [31]. We also filtered and sequenced four variants on three genes in these regions, but the variants did not co-segregate with the TEGI phenotype in the respective families (Table S14a–e). We note that other phenotypes were linked to these regions, e.g., both the receptive vocabulary and omnibus phenotypes were linked to a region of markers on chromosome 6. Early LIs are predictive of later RDs [54,55], such that low language performance at a young age (when the TEGI is administered) was likely correlated with the other phenotypes. This may mean that the combination of low performance on the TEGI and low performance on other phenotypes was likely driving the linkage. The focus on only TEGI performance at the WES variant filtering level and the reduced number of WES families may have limited the power of the regions to target variants of interest. However, the lack of variants of interest in the RD regions previously linked to the TEGI does not suggest that targeted investigation of related phenotypes is not of value. Findings from related phenotypes should always be considered, especially given that common causal pathways may be identified through this consideration and that targeted investigation adds confirming and disconfirming evidence for existing reports.

Across the nine genes of interest, *FURIN*, *BAHCC1*, *NOL6*, *FLNB*, and *KMT2D* show higher brain expression than *PCDHB3*, *IQGAP3*, *PDHA2*, and *GLI3* [56]. We present supporting evidence for *PCDHB3* and *NOL6* according to their functions and previous reports.

The protein product of *PCDHB3* is protocadherin beta 3. Protocadherins are a cadherin subfamily composed of three gene clusters, PCDH-α, PCDH-β, and PCDH-γ [57]. Cadherins and protocadherins are a large family of proteins involved in diverse functions, like hearing, balance, and neurodevelopmental and neurological processes among mammals [57]. The highest expression of these genes was observed in the nervous system [57]. Beta protocadherins localize to the synapse junctions during early development in mice, demonstrating their significance of neuronal connections in the mammalian nervous system [58]. Interestingly, the genetic investigation of a male child with severe non-syndromic language delay showed an intergenic deletion (220 Kb) at the homologous region of chromosomes X and Y spanning PCDH11X/Y [59]. A genetic study of another child with a sexual developmental disorder with severe language impairment and autistic behavior reported a concurrent deletion of PCDH11Y and NLGN4Y, indicating the role of protocadherin in developing even syndromic language impairment [60]. Another genetic study of a multiplex family with dyslexia observed ancestral genetic variations in PCDHG, showing the role of developing reading skills in humans [61]. More interestingly, the broad specificity of the antibodies to the isoforms was localized to the cortical areas related to language, indicating the significance of the splicing mechanisms in brain tissues for regulating the posttranscriptional regulation of protocadherins and other necessary transcripts for their diversity in the brain tissues [19,62]. We speculate that such family-based studies provide an excellent opportunity to replicate and discover new gene targets involved in language acquisition.

*NOL6* (nucleolar protein 6) is essential in the biogenesis of ribosomes. Ribosomes are an integral component of protein synthesis in all cells. The ribosomal RNAs and proteins complete the biogenesis of ribosomes in the nucleolus. The translational efficiency of ribosomes is determined by several factors, including ribosome assembly and how the mRNAs load to the ribosomes. This translational efficiency is variable in the complex neuronal structures creating variability in the protein expression [63]. Deficits in ribosome biogenesis can result in multiple neurodevelopmental disorders. Neuronal cell types and the developmental period in which the deficiency was experienced determine the pathological consequences of these deficits [64]. We identified likely pathogenic variants in *NOL6* in multiple families, leading to our prediction that it is involved in gene pathways suggested in language impairment.

### 4.1. Limitations

A few key limitations of the current study should be noted: the lack of a grammar phenotype in parents and the possible missingness due to using WES versus whole-genome sequencing (WGS). The family-based approach was limited by the lack of a grammar phenotype in parents and any children who entered the study after the age 8; 11 years. However, given that rare variants of interest were identified without parent grammar phenotypes, we predict a study including grammar phenotypes in parents would be even more powerful. It is always important to consider possible missingness due to the genetic method utilized. Variants called by WES and WGS have been compared, showing about 3% of coding variants present in the WGS output were not present in the WES output [65]. This means additional coding variants segregating with the TEGI could have been missed due to low coverage and higher false positive call rate in the WES vs WGS.

### 4.2. Future Directions

In the future, grammar phenotypes capturing grammar at the same precise level as the TEGI should be utilized for older ages. Behavioral evidence suggests that such precise measurement is possible. In adolescence, at 15 and 16 years old, measurement of correct and incorrect grammatical judgments of questions where ‘be’ and ‘do’ were omitted is specific for the extended optional infinitive phenotype and shows high heritability in twins [4,66]. 

Additionally, future study should sequence all coding regions of genes prioritized for sequencing in the additional probands. Additional criteria may need to be considered before determining how many of the genes should be sequenced in full. Other criteria could include the expression data compiled from databases and provided in the current report, or additional expression data from BrainSpan about expression in the fetal brain [67]. Testing the larger proband group for additional rare variants in the novel candidate genes would allow for gene level testing of the rate of rare missense or loss of function variants. While variant level comparison can be informative, it can also be dependent on multiple factors of the downstream analysis, as shown by the recent WES investigations of family 5886 and 4075. Gene level significance testing provides stronger evidence for suggesting a candidate gene for a disorder, which should be considered carefully. However, the importance of variant level significance and a variant’s possible deleterious role within their given causal pathway should not be disregarded in favor of gene-level significance. 

In the long term, the newly suggested genes in this study can be helpful in determining where to look in neuroimaging for differences in groups with and without SLI. For example, the suggested genes can be further queried in gene pathway databases. One such database is STRING db, which assesses functional protein association networks [68]. Using the example of *NOL6*, STRING output shows strong connections to 10 other genes, all interconnected. The genes in the associated pathways could be checked in the existing WES output for variants and brain expression of these genes could be evaluated to further narrow the search for causal pathways of language acquisition and disordered language acquisition.

### 4.3. Implications of Family-Based Genetic Study for Understanding Factors Involved in Language Development

Long-standing questions surrounding the rapid acquisition of language by humans (i.e., adult-like language by age five in most individuals) have prompted investigations from multiple perspectives and an ongoing debate concerning the extent to which language ability is inherited [2,69,70]. Behavioral genetic studies of twins and families first showed the significant role of genetics in language acquisition and molecular genetic studies followed [1,2,3,4,5,6,18,19,30,32,33]. The current study added to the growing literature of molecular genetic studies by specifically targeting genetic influences on abstract shared grammar scaffolding under the assumption that humans have a specific universal aptitude toward language, such that our findings could contribute to the larger discussion of the role of genetic and environmental factors at play specifically in children’s rapid early acquisition of complex structures in English. The investigation targeted individuals with SLI showing low performance on the TEGI, which measures a deficit in tense and agreement production or finiteness marking in English grammar [15]. 

Using performance on the TEGI ensured a precise phenotype. Precise phenotyping is required for precise genetic investigation and this study adds to the literature with a test of a precise phenotype that consistently shows significant heritability estimates [3,4,5,30]. The significant heritability estimates reported in 16-year-old twin pairs who completed a grammaticality judgment task indicates that the influence of genetics in grammar remains past the age measured by the TEGI [4]. Additionally, a recent report of individuals with SLI, which includes those from the current study and showed that the same pattern of difficulty in finiteness marking extends through age 18 when participants complete age-appropriate more challenging tasks concerning their understanding of complex linguistics structures [29]. Both results in older age groups support the importance of precisely defining this grammatical impairment in behavioral and genetic research.

The current study cross-referenced previously reported candidate genes identified from studies of individuals with SLI and related phenotypes. Similarly, the genes identified from our family-based investigation provide additional targets for future studies of larger cohorts of unrelated individuals, which are the focus of many other groups studying the genetics of language [21,22,23], just as the foundational findings of linkage to chromosome 16q and 19q to SLI have been continually cross-referenced in results from both families and larger and larger cohorts of individuals with language and reading phenotypes [21,36,51,71,72].

Our focus on possible genetic contributions to SLI recognizes the full complexities of possible causal pathways. Children’s language acquisition unfolds in the context of social and cognitive dimensions of development, along with other health-related factors. Identification of possible genetic pathways can identify sources of individual variance that further our understanding of individual differences that influence language development, which could aid in individualizing implementation of effective therapeutic approaches, which may include parent counseling, specialized peer social settings, focus on the cognitive underpinnings of vocabulary development, and other elements of a comprehensive intervention approach. Socioeconomic status is one such individual difference that is commonly considered in studies of language acquisition [73,74,75,76].

Children with SLI can be confused with children from low-income families, where “low income” is an indirect index of familial social resources for young children. Numerous studies found that children in families with lower-than-average incomes could be delayed in language acquisition [74,77]. For example, vocabulary development is predicted by maternal education in our longitudinal studies of vocabulary in children with and without SLI [78]. The design of this family-based study of SLI allowed us to explore this possible association, using maternal education as a proxy for family social resources in a total of 191 child participants, *n* = 175 SLI affected and *n* = 16 unaffected child family members in the eight discovery pedigrees (i.e., families selected for predominance of affectedness). In this supplemental analysis, children were grouped according to affectedness status on their omnibus language performance at entry to the study (consistent with the proband entrance criteria reported in the Methods and previous publications [18,19,31]). Maternal education was scored on a scale of 1 = some high school, no diploma, 2 = high school graduate diploma or GED, 3 = some college, no degree, 4 = bachelor’s degree, 5 = some graduate studies, and 6 = graduate degree. Thus, if low social resources, as indexed by maternal education, were driving the high proportion of affected children, we would expect low levels of maternal education in the SLI group. The means on the education scale were the unaffected group *M* = 2.94 (SD = 1.00) and SLI affected group, *M* = 3.03 (SD = 1.28). An independent samples *t*-test that accounted for variance at the family level showed no statistically significant group differences in maternal education: *t* (42.7) = −1.33, *p* = 0.190, indicating that the findings from our sample do not support a close association of maternal education and SLI.

## 5. Conclusions

Our family-based investigation prioritized multiple genes not previously suggested for SLI based on the sharing of rare variants between unrelated individuals affected by the TEGI. These findings indicate the TEGI phenotype has the potential to play a vital role in the future genetic inquiry of SLI. More broadly, focusing on the TEGI phenotype at the genetic level can inform causal pathways involved in language acquisition and the genetic underpinnings of brain structures uniquely provided to humans.

## Figures and Tables

**Table 1:** Additional Information for Variants Tested in the Probands (*n* = 146).

Gene	Discovery Pedigree (s)	Additional Probands Carrying Variant (s)		Fisher’s Test *p*-Value ^2^	rsID	AA Change	MAF	# of Damaging In Silico Scores ^3^	AA Change (HOPE)		Causality Prediction
		Affected	Unaffected						Size	Charge	
*PDHA2*	5463, 5886	2 ^	0	0.3590	rs147966234	pArg286Pro	0.0089 ^1a^	5/5	∨	POS to neu	P
*PCDHB3*	4093, 4130	1	0	0.5112	rs61739886	p.Thr81Ile	0.0064 ^1a^	3/5	∧	NCC	P
*FURIN*	4075, 5886	0	0	0.06445	rs150925934	p.Arg462Trp	0.0017 ^1a^	4/5	∧	POS to neu	P
*NOL6*	4130	1	0	<0.0001 *^,1,4^	rs114465306	p.Pro134Leu	0.00008 ^1^	4/5	∧	NCR	P
	5931	0	0	NA	rs114110943	p.His366Tyr	0.006 ^1b^	2/5	∧	NCR	Likely P
*IQGAP3*	4093	0	0	NA	rs147754283	p.Arg630Trp	0.00005 ^1a^	3/5	∧	POS to neu	Likely P
	5886	2	0	0.1094	rs112144116	p.Ala562Thr	0.0034 ^1a^	4/5	∧	NCC	P
*BAHCC1*	4093	0	0	NA	rs369588790	p.Arg2199Gln	0.00006 ^1a^	2/5	NCR	NEG to neu	Likely P
	5931	0	0	NA	rs200719992	p.Gln2463Glu	0.0066 ^1b^	4/5	∨	POS to neu	Likely P
*GLI3*	4093, 4130, 4132	6 ^	4	0.7946	rs35364414	p.Arg1537Cys	0.0536 ^1a^	4/5	∨	POS to neu	B
*FLNB*	4132	2 + 1 !	0	0.3398	rs116826041	p.Ile2319Thr	0.0093 ^1a^	3/5	∨	NCR	B
*KMT2D*	5463	0	0	NA	rs146044282	p.Asp3419Gly	0.0015 ^1a^	3/5	∨	NCR	Likely P

Note. * significant, ^1^ global MAF, ^1a^ Subpop = non-Finnish European MAF, ^1b^ Subpop = African MAF (all from gnomAD v2.1.1.; ^2^ Fisher’s Exact Test for Count Data compared allele count in unrelated probands (including discovery pedigree probands) to appropriate subpopulation gnomAD MAF; ^3^ # of damaging in silico scores include: SIFT, Poly-Phen2, MutationTaster, PROVEAN, and Mutation Assessor; ^4^ Unreadable sequences for 35 probands in nucleotides surrounding the variant; ^ same proband represented, ! Additional discovery proband (M4299 in family 4379; WES output showed that the variant was called in the proband, M4299, and their sibling who was unaffected on the TEGI (M4304) and therefore not prioritized under filtering workflow 2); AA = amino acid; HOPE (Have yOur Protein Explained) output: ∧ = size of AA increased, ∨ = size of AA decreased, + = more hydrophobic, NCR = no change reported, Charge change: POS = positive, neu = neutral, NEG = negative, NCC = no change in charge, NCR = no change reported; Causality classifications: pathogenic (P) = (1) MAF < 0.05 (in gnomAD v2.1.1 exomes) AND (2) EITHER co-segregating OR carried by >1 proband OR some significant change to amino acid structure AND (3) positive GERP score (conserved) AND (4) CADD Phred score ≥ 20 AND (5) ≥2 damaging in silico prediction scores (of those analyzed ^3^) AND (6) a change in size or charge in the amino acid according to HOPE output; benign (B) = missing one of the 6 classification criteria All in silico scores were acquired using the hg19 locations prior to 15 April 2022. I converted the hg38 locations using the ‘Lift Genome Annotations’ tool within UCSC Genome Browser [53].

## Data Availability

We have provided parts of the dataset generated during and/or analyzed for our study in the Supplemental Material. Additional parts are available from the corresponding author on reasonable request.

## Supplementary Materials

The following supporting information can be downloaded at: https://www.mdpi.com/article/10.3390/children10071119/s1, Table S1: Candidate Gene List (*n* = 113, based on a combination of reviews); Table S2: Distribution of Affectedness Across the Four Possible Phenotypes in the Whole exome Sequenced Individuals (*n* = 34); Table S3: Distribution of Affectedness Across the Four Possible Phenotypes in the Additional Probands (*n* = 146); Table S4: Family-specific Criteria for ‘family-specific variant comparison lists’; Table S5: Family-specific Criteria applied for ‘co-segregating variant lists’; Table S6: Primers used for confirmation via sequencing; Table S7: Number of Rare Variants: Familywise Filtering Workflow 1; Table S8: 47 Genes Resulting from Cross-Referencing of ‘Family-Specific Variant Comparison Lists’; Table S9: Summary of 44 Variants on 23 Genes Prioritized from Filtering Workflow 1 Cross-referenced List; Table S10: Summary of Prioritized Variants from Filtering Workflow 1 Sequenced and Confirmed in Family Members; Table S11a: 18 Rare Variants Prioritized in Family 4093 Shared by All Four Family Members Affected on the TEGI; Table S11b: 31 Rare Variants Prioritized in Family 4130 Shared by Both Family Members Affected on the TEGI; Table S11c: 31 Rare Variants Prioritized in Family 4132 Shared by Two or More Family Members from Different Branches Affected on the TEGI; Table S11d: 37 Rare Variants Prioritized in Family 4075 Shared by Three or More of the Four Family Members Affected on the TEGI. Table S11e: 17 Rare Variants Prioritized in Family 4379 Shared by Two or More of the Three Family Members Affected on the TEGI; Table S11f: 6 Rare Variants of Large Effect Prioritized in Family 5463 Shared by All Three of the Four Family Members Affected on the TEGI; Table S11g: 23 Rare Variants Prioritized in Family 5886 Shared by All Four of the Four Family Members Affected on the TEGI; Table S11h: 18 Rare Variants Prioritized in Family 5931 Shared by Both Family Members Affected on the TEGI; Table S12: Number of Candidate Gene Variants: Familywise Filtering Workflow 2b; Table S13: Summary of Confirmation Notes for the 13 Variants Prioritized from Filtering Workflow 2 Output Sequenced in Family Members; Table S14a: Number of Variants on Chromosome 3q13.12-q13.31: Familywise Filtering Workflow 2a; Table S14b: Variants prioritized familywise within RD region: chr3q13.12-q13.31, previously significantly associated with TEGI phenotype; Table S14c: Number of Variants on Chromosome 6p21.1-p22.3: Family Filtering Workflow 2a; Table S14d: Variants prioritized familywise within RD region: chr6p21.1-p22.33q13.12-q13.31, previously significantly associated with TEGI phenotype; Table S14e: Primers used for confirmation of variants in RD regions via sequencing.
