# Supplementary material for: Innovative Family-Based Genetically Informed Series of Analyses of Whole-Exome Data Supports Likely Inheritance for Grammar in Children with Specific Language Impairment
Source: Children (Basel). 2023 Jun 28;10(7):1119. doi: 10.3390/children10071119 (PMC10378399; doi:10.3390/children10071119)
Supplement: Supplementary file 1 [file children-10-01119-s001.zip › children-2428542-supplementary.pdf]

Table S1.

Candidate Gene List (n = 113, based on a combination of recent reviews).

Gene	Phenotype associated	Review article or original article
ABCC13	DLD/SLI	Chen et al., 2017; Mountford et al., 2019
ANKK1	SSD	Guerra et al., 2019
ANKR12	CAS	Guerra et al., 2019
AP4E1	ST	Guerra et al., 2019
APOE	AP (aphasia)	Guerra et al., 2019
ARHGEF39	DLD/SLI	Mountford et al., 2019
ARL17	DL	Guerra et al., 2019
ATP13A4	CAS	Guerra et al., 2019
ATP2C2	DLD/SLI, ASD	Chen et al., 2017; Guerra et al., 2019; Mountford et al., 2019; Mountford et al., 2022
AUTS2	ASD	Chen et al., 2017; Mountford et al., 2019
BCL11A	CAS with expressive language and mild language delay	Mountford et al., 2019; Mountford et al., 2022
BDNF	SSD	Guerra et al., 2019
*BIRC6**	DLD/SLI	Chen et al., 2017
C2ORF3	DL	Guerra et al., 2019
CDH18	CAS	Guerra et al., 2019
CHD3	CAS, DLD	Guerra et al., 2019; Mountford et al., 2019; Mountford et al., 2022
CHRNA3	SSD	Guerra et al., 2019
CMIP	DLD/SLI, DL, ASD	Chen et al., 2017; Guerra et al., 2019; Mountford et al., 2019
CNTNAP1	CAS	Guerra et al., 2019
CNTNAP2	DLD/SLI, CAS, ST, DT, ASD	Chen et al., 2017; Guerra et al., 2019; Mountford et al., 2019; Mountford et al., 2022
CNTNAP5	ASD, DL	Chen et al., 2017; Mountford et al., 2019
COL4A2	DLD/SLI	Guerra et al., 2019
COMT	DL	Guerra et al., 2019
CTTNBP2	ASD	Guerra et al., 2019
CYP19A1	SSD	Guerra et al., 2019
DCDC2	SSD, DL	Chen et al., 2017; Guerra et al., 2019; Mountford et al., 2019
DGK1	DL	Guerra et al., 2019
DOCK4	ASD, DL	Chen et al., 2017; Mountford et al., 2019
DRD2	SSD	Guerra et al., 2019
DYM	DL	Guerra et al., 2019
DYX1C1	DL, SSD	Chen et al., 2017; Guerra et al., 2019; Mountford et al., 2019
DYX2	DL, ASD	Guerra et al., 2019
DYX3	DL	Guerra et al., 2019
DYX5	DL, SSD	Guerra et al., 2019
DYX6	DL	Guerra et al., 2019
DYX8	DL, SSD	Guerra et al., 2019
DYX9	DL	Guerra et al., 2019
ELKS	CAS	Guerra et al., 2019
ELP4	SSD	Guerra et al., 2019
EN2	ASD	Guerra et al., 2019
ERC1	CAS	Chen et al., 2017; Mountford et al., 2019; Mountford et al., 2022
*FAT3**	DLD/SLI	Chen et al., 2017
FLCN	CAS	Guerra et al., 2019
*FLNB**	DLD/SLI	Chen et al., 2017
FLNC	DL	Mountford et al., 2019

Gene	Phenotype associated	Review article or original article
FOXP1	CAS, DT, DLD/SLI, ASD	Chen et al., 2017; Guerra et al., 2019; Mountford et al., 2019;
FOXP2	CAS, SSD	Mountford et al., 2022
GCFC2	ASD	Guerra et al., 2019
GLI3	AP (aphasia)	Guerra et al., 2019
GLP2R	CAS, ASD	Guerra et al., 2019
GNPTAB	ST	Guerra et al., 2019
GNPTG	ST	Guerra et al., 2019
GRIN2A	focal epilepsy with speech disorder, with or without ID	Chen et al., 2017; Mountford et al., 2019; Mountford et al., 2022
GRIN2B	ID, ASD	Chen et al., 2017; Mountford et al., 2019
GRN	AP (aphasia)	Guerra et al., 2019
HRAS	ASD	Guerra et al., 2019
*IDO2^*	DLD/SLI	Chen et al., 2017
KAT6A	CAS	Guerra et al., 2019; Mountford et al., 2019; Mountford et al., 2022
KIAA0319	DL, SSD, DLD/SLI, CAS, ASD	Chen et al., 2017; Guerra et al., 2019; Mountford et al., 2019
*KIAA0586**	DLD/SLI	Chen et al., 2017
KIAA1267	DL	Guerra et al., 2019
*KMT2D**	DLD/SLI	Chen et al., 2017
LRRC37A	DL	Guerra et al., 2019
MC5R	DL	Guerra et al., 2019
MED13L	ASD	Guerra et al., 2019
MET	ASD	Guerra et al., 2019
MKL2	CAS	Mountford et al., 2019
MRPL19	DL	Guerra et al., 2019
*MUC6^*	DLD/SLI	Chen et al., 2017
MYO10	CAS	Guerra et al., 2019
*MYO16**	DLD/SLI	Chen et al., 2017
*MYO19**	DLD/SLI	Chen et al., 2017
NAGPA	ST	Guerra et al., 2019
NBEA	ASD	Guerra et al., 2019
NCOR1	CAS	Guerra et al., 2019
NDST4	DLD/SLI	Guerra et al., 2019; Mountford et al., 2019
NEDD4L	DL	Guerra et al., 2019
NEK8	CAS	Guerra et al., 2019
NFXL1	DLD/SLI	Chen et al., 2017; Guerra et al., 2019; Mountford et al., 2019; Mountford et al., 2022
NIPBL	CAS	Guerra et al., 2019
NOP9	DLD/SLI	Guerra et al., 2019; Mountford et al., 2019
NSF	DL	Guerra et al., 2019
NSFP1	DL	Guerra et al., 2019
*NUDT16L1^*	DLD/SLI	Chen et al., 2017
OR52B2^*	DLD/SLI	Chen et al., 2017
*OR6P1^*	DLD/SLI	Chen et al., 2017
*OXR1^*	DLD/SLI	Chen et al., 2017
*PALB2**	DLD/SLI	Chen et al., 2017
PAX6	SSD	Guerra et al., 2019
PCDH11X	DL	Guerra et al., 2019
PSEN1	AP (aphasia)	Guerra et al., 2019
PTEN	ASD	Guerra et al., 2019
RBFOX2	DLD/SLI with DL	Guerra et al., 2019; Mountford et al., 2019
ROBO1	SSD, DL, ASD	Chen et al., 2017; Guerra et al., 2019; Mountford et al., 2019

Gene	Phenotype associated	Review article or original article
ROBO2	DLD/SLI, DL	Guerra et al., 2019; Mountford et al., 2019
*SCN9A**	DLD/SLI	Chen et al., 2017
SEMA6D	DLD/SLI	Chen et al., 2017; Mountford et al., 2019
SETBP1	CAS, DLD/SLI	Chen et al., 2017; Guerra et al., 2019; Mountford et al., 2019; Mountford et al., 2022
SETD1A	CAS	Guerra et al., 2019; Mountford et al., 2019; Mountford et al., 2022
SETX	CAS, DT	Guerra et al., 2019
SMCR8	CAS	Guerra et al., 2019
SRPX2	DLD/SLI, ASD, Rolandic seizures and ID	Chen et al., 2017; Guerra et al., 2019; Mountford et al., 2022
*STARD9**	DLD/SLI	Chen et al., 2017
*SYNPR^*	DLD/SLI	Chen et al., 2017
TDP-43	AP (aphasia)	Guerra et al., 2019
TM4SF20	DLD/SLI	Mountford et al., 2019; Mountford et al., 2022
TNRC6B	CAS	Guerra et al., 2019; Mountford et al., 2019; Mountford et al., 2022
TTRAP	DL	Guerra et al., 2019
WDR5	CAS	Guerra et al., 2019; Mountford et al., 2019; Mountford et al., 2022
ZFHX4	CAS	Guerra et al., 2019; Mountford et al., 2019; Mountford et al., 2022
ZGRF1	CAS	Guerra et al., 2019
ZNF385D	DLD/SLI	Guerra et al., 2019
ZNF277	DLD/SLI	Ceroni et al., 2014; Mountford et al., 2022

Note. *genes with more than 1 variant in a single SLI proband from the SLI Consortium (not from Chen et al., 2017 list of candidate genes), ^genes with a stop gain variant in SLI proband from the SLI Consortium (not from Chen et al., 2017 list of candidate genes), we have also noted any genes referenced in a more recent review (Mountford et al., 2022), DLD/SLI-developmental language disorder/specific language impairment, DL-dyslexia, CAS-childhood apraxia of speech, ST-stuttering, SSD-other speech sound disorders, AP-aphasia, DT-dysarthria, and ASD-autism spectrum disorder

Table S2.
Distribution of Affectedness Across the Four Possible Phenotypes in the Whole exome Sequenced Individuals (n = 34).

Total	TEGI affected status totals	TEGI composite	TEGI screener	Omnibus	PPVT
19	23 Composite & screener AFF	-	-	-	-
1		-	-	-	+
2		-	-	+	-
1		-	-	+	+
2	4 composite AFF only	-	+	-	+
2		-	+	-	-
1	1 only screener avail AFF	missing*	-	-	+
1	6 UNAFF on TEGI composite + screener	+	+	-	-
2		+	+	-	+
1		+	+	+	-
2		+	+	+	+
34	34	27/33* AFF	24/34 AFF	28/34 AFF	25/34 AFF

Note. *proband (ID#: M3287). TEGI affectedness was based on a Z score that was calculated using the data provided in the test manual. A child with a Z score ≤ -1 was considered affected on the TEGI.

Table S3.

Distribution of Affectedness Across the Four Possible Phenotypes in the Additional Probands (n = 146).

Total	TEGI affected status totals	TEGI composite	TEGI screener	Omnibus	PPVT
77	108 Composite & screener AFF	-	-	-	-
24		-	-	-	+
3*		-	-	+	-
4*		-	-	+	+
9	15 Composite AFF only	-	+	-	-
5		-	+	-	+
1*		-	+	+	-
2	2 Screener AFF only	+	-	-	-
11	21 UNAFF on TEGI composite + screener	+	+	-	+
10		+	+	-	-
146	146	123/146 AFF	110/146 AFF	138/146 AFF	102/146 AFF

Note. *Given the primary interest in morphosyntax in the larger longitudinal project, entrance criteria were sensitive to the possibility that some children would have low morphosyntax abilities but perform better on an omnibus standardized language measure. Therefore, eight of the children were entered on the basis of their low performance on TEGI, despite their average performance on an omnibus standardized language measure.

Table S4.
Family-specific Criteria for 'family-specific variant comparison lists.'

Family	Family-specific criteria
4093	Removed: variants shared by both parents<br>Removed: variants M3330 (persistently low NV-IQ) shared with three or fewer of the other siblings (all affected on the TEGI)
4130	No family-specific criteria for 'family-specific variant comparison lists'
4132	Removed: variants only shared by individuals in one branch (exception: no GTs called in one branch – accounts for subtle differences in the two rounds of WES)<br>Removed: variants shared by > 5 of the family members with an unknown TEGI phenotype
4075	Removed: variants shared by both parents and the half-sibling (M3062)
4379	Kept: only variants with a 0/0 WT genotype in M4304 (unaffected on the TEGI)
5463	Removed: variants shared by both siblings unaffected on the TEGI (M8335, M7769)<br>Removed: variants shared by both parents
5886	Removed: variants shared by > 4 of the family members with an unknown TEGI phenotype
5931	Removed: variants shared by > 3 of the family members with an unknown TEGI phenotype (A093 considered unknown – removed from WES filtering due to persistently low NV-IQ)<br>Removed: variants shared by both siblings unaffected on the TEGI (A0981, A0982)

Table S5.
Family-specific Criteria applied for 'co-segregating variant lists.'

Family	Family-specific criteria
4093	Kept: variants shared by all four siblings affected on the TEGI
4130	Kept: variants the two siblings share with their mother (mother showed low performance on a standardized omnibus language measure)
4132	Removed: variants not shared by the proband (M3387) and their child (M4950), both are affected on the TEGI
4075	Kept: variants shared by at least three of the four siblings affected on the TEGI
4379	Removed: variants not observed in the proband's cousin (M8841), who is affected on the TEGI
5463	Removed: variants observed in either sibling unaffected on the TEGI (M8335, M7769)
5886	Kept: variants shared by all four siblings affected on the TEGI
5931	No family-specific criteria for 'co-segregating variant lists'

Table S6.

Primers used for confirmation via sequencing.

Family	Prioritization Workflow	Gene	Chr	Variant Location (hg38)	Forward Primer	Reverse Primer	PCR product size	Optimized Tm	Direction for seq
				81461957	CCACACCCATATTTGGCAAC	gtttccttgggaaccaggag	996*	54°+DMSO	
4093		*BAHCC1*	17		CCACACCCATATTTGGCAAC	CTTCTTGTCCTTGCCCACAA	338^	55°	Rev
5931				81461259	TGCTTCCTGTCCAGGGCCAC	gagagggaagggcagcaggg	300	61°+ DMSO	Fwd
4093		*GPT*	8	144506908	cacagGAGAAGCAGGCAGTG	ctcctgagcacacagtccag	555	Not optimized	NA
5886				144506631					NA
4093				75625226	gcctctagagccaccaactg	gtgcctattgtgggcaagac	395	56°	Fwd
4132		*MYO15B*	17	75589672	AGCGTTGCGCCTGGCTGGCT	ACGAGGCCAACCTCGGCCTTCCC	247	58°+ DMSO	Fwd
4075				75621145	ggcaccaggtttcttgtgat	caggcagggagagtgctaag	277	Not optimized	NA
				75619800	cttcctgtagGGCGAGAGTG	tcgctcttcagtgacagctc	274	56°	Rev
4093, 5886		*PCDH12*	5	141945390	CGGGAGGACCCTCAGTTTAG	cagtcaccctaaagttctcagg	250	56°	Fwd
4093, 4130		*PCDHB3*	5	141100891	TGTGGCTGAGGAAAAAGAGAA	TTGCAAAGGGTTTTGCAGTA	244	56°	Rev
5463, 5886		*PDHA2*	4	95841007	AGCAGCAGCCAGCCCTGATTAC	CCACAGTGGCGAGCTTGCTGT	293	56°	Fwd
4093, 4132		*RBM26*	13	79344671	aggatatcatgaacggctga	cagtggcagccaacaaacac	299	56°	Rev
4132, 4075, 4379, 5886	Whole-exome wide	*SYCP1*	1	114994979	ccaccagcttctcatctttgt	tttggctctggcaaataagaa	352	56°	Rev
4130, 4379		*ZNF226*	19	44176298	TGGGAGAAAAACTTAAGTGTGATG	TGAGCTACAAATGAAGCCCTTA	389	56°	Fwd
4093		*HS6ST1*	2	128318170	CCCCACTACGAGAAGAAGTA	gaagagagtgagagcgaggt	568	53°+ DMSO	Fwd
4130, 4075				128318223					Rev
4093		*IQGAP3*	1	156551755	tcagaagggtgattttgtgt	caagcagaaaaggttcagag	250	56°	Fwd
5886				156548686	ctctttcttagGGGTCCTTG	cagactgggtacagtgcaac	360	56°	Rev
4132				75857098	attctcacatgacctttggt	GGATGATGTTTCAAAAGGAA	250	56°	Rev
5463		*MYO6*	6	75830425	cattgttgtgcaacagaagaaa	GCCTTTCCTCCTCTTCCTTT	288	56°	Fwd
4379				75890140	gatcagggaatactcagggaat	cCAATTGCAAAGACATGAGG	264	56°	Rev
4130		*NOL6*	9	33468803	gggaagattgggagtttggt	CATTGATGTCTGGTCGGATG	249	56°	Fwd
5931				33470169	tacctgagcgcctgtgtgat	ctgctactgggatgagagga	272	56°	Fwd
4075, 5886		*FURIN*	15	90880107	tgcatcatcgacatcctcac	cagggacagagcaagcacCT	336	56°	Fwd
4132, 5886		*PAK2*	3	196803031	aaacctacactcaaaagattgg	AGCTCAGATATTTCTGCTTCA	249	56°	Fwd
4132, 5463, 5886		*TMBIM4*	12	66138156	acttgccttgcctttttctg	GGCTGATGGCAGCTAATACG	295	56°	Rev

Family	Prioritization Workflow	Gene	Chr	Variant Location (hg38)	Forward Primer	Reverse Primer	PCR product size	Optimized Tm	Direction for seq
4093, 4130, 4132	Previously reported Candidate Genes (n = 113)	*GLI3*	7	41964464	GGTGACAAGCACAGTGGACA	tttttcctaaagcCTATTGCAT	299	56°	Rev
4093		*ZFHX4*	8	76856262	ATTGAGATCCTGTCGGATGC	ctgggccagtaaggactctg	286	56°	Fed
4132		*SCN9A*	2	166307016	ggtaaactgctgatattgatgtga	ccagagtctttcaaggtgcaa	298	56°	Fwd
5886				166288642	aaaatttgaaagtgagcaaagga	TCAATGTTTGCCTGGTTCTG	450	56°	Rev
4132		*FLNB*	3	58159621	cacccacagttttgggtagg	ctcccttgggacacacattt	298	56°	Rev
4132		*GRIN2B*	12	13611737	aaccctgagcactgcttttt	cacctgagggttccttttca	283	56°	Fwd
4132		*GRIN2A*	16	9763667	GGATGGGGAACCTCTATGAC	TTTTCCCCGAGAGTTTGCTT	297	56°	Rev
4379		*FAT3*	11	92354793	CTTGAAAGTTCAGGCATTGG	GCTACTGGGAATGAAAGCTG	300	56°	Rev
5463		*COL4A2*	13	110491254	gcctctctccattcctgaag	tgttgaccgcctttgttctg	300	56°	Fwd
5463		*KMT2D*	12	49034911	tggctcctaattcagtggtctt	aaagttggaaagcaaagagacg	370	56°	Fwd
5886		*CHRNA3*	15	78601954	CGGTTCCTGGTCCTACGATA	ATGCACAGGGTCACCTTCTC	300	56°	Rev
4130, 4132		*NOP9*	14	24300643	GTTGGGATTCAGTCCCTTGA	AGAGTTCTGACCACGAAGCTG	291	56°	Fwd

Note. *primer pair used to initially amplify genomic region, ^primer pair used internally to amplify prioritized variant

Table S7.

Number of Rare Variants: Familywise Filtering Workflow 1.

	**4093**	**4130**	**4132**	**4075**	**4379**	**5463**	**5886**	**5931**
Total number of variants	21,592	12,752	47,401	31,785	30,607	27,345	34,292	34,714
Keep: exonic, splicing, exonic; splicing	6,722	6,360	14,381	8,146	7,639	6,979	7,050	18,157
Remove: synonymous	4,435	4,002	9,115	5,263	5,117	4,639	4,689	10,180
Remove: segmentally duplicated	3,069	2,526	6,841	3,512	3,538	3,283	3,177	7,701
Keep: gnomAD subpop[1] MAF < 0.01	2,134[1a]	1,452[1a]	4,634[1a]	2,045[1a]	2,670[1a]	2,333[1a]	2,139[1a]	3,749[1b]
Keep: <u>variants shared by 2+ individuals with TEGI phenotype</u>	428	229	688	361	226	217	515	102
Keep: CADD pred score ≥ 20	254	189	423	218	125	187	297	67
Keep: positive GERP score	245	108	411	215	123	185	292	65
Remove: bioinformatic prediction score sums[3] = 0/5, 1/5, 0/4, 1/4, 0/3	209	105	346	172	102	150	234	50
Apply: family-specific criteria[2] (# of unique genes) = 'family-specific variant comparison lists'	111 (101)	80 (72)	110 (99)	169 (152)	48 (37)	97 (85)	208 (192)	18 (18)
Total number of shared genes (4+ individuals affected on TEGI measure across at least 2 families have a variant on a gene) = 55								
3 genes on X chr (*RBMX*, *RPL10*, *MAGEC1*)								
2 genes – variants have low quality and previous attempts to confirm for other projects was unsuccessful (*AK2*, *PABPC1*)								
1 gene is so physically large it is more likely to have mutations, regardless of phenotype (*TTN*)								**47 genes of interest remain**
2 genes from gene families that commonly show up in filtered WES output, regardless of phenotype (*MUC3A*, *ANKLE1*)								**(listed in full in Table S8)**
Apply: family-specific co-segregation criteria[4] = 'co-segregating variant lists' (listed in full in Tables A 2 – A 9)	18	31	31	37	17	6	23	18
Genes from cross-referenced list[2]	1	6	5	5	4	0	5	2
Total variants on genes from cross-referenced list								28
Total unique variants on genes from cross-referenced list								25
								**23 genes of interest remain (Table S9)**

Note. MAF = minor allele frequency; GERP = Genomic Evolutionary Rate Profiling; CADD = Combined Annotation Dependent Depletion; [1a]Subpop = non-Finnish European, [1b]Subpop = African, [2]Detailed in Table 6, [3]Prediction score sum includes = SIFT, PolyPhen-2, Mutation assessor, PROVEAN, MutationTaster2, [4]Detailed in Table 7

Table S8.
47 Genes Resulting from Cross-Referencing of 'Family-Specific Variant Comparison Lists'.

		Family								
**Chr**	**Gene**	**4093**	**4130**	**4132**	**4075**	**4379**	**5463**	**5886**	**5931**	**Notes**
5	*ABLIM3*	X					X			Only on Comparison Lists
8	*ADAM28*							X		Only on Comparison Lists
16	*ADCY7*	X					X			Only on Comparison Lists
5	*AP3S1*	X			X					Only on Comparison Lists
17	*BAHCC1*	X							X*	
19	*BICRA*					X*	X	X		
20	*BPIFB4*	X		X						Only on Comparison Lists
12	*DNAH10*						X	X		Only on Comparison Lists
2	*DYSF*						X	X		Only on Comparison Lists
**6**	***FGD2***			X	X					
15	*FURIN*				X*			X		Only on Comparison Lists
8	*GPT*	X						X*		
2	*HS6ST1*	X	X*		X*					
1	*IQGAP3*	X						X*		
1	*KIAA0040*	X	X		X		X			Only on Comparison Lists
4	*LARP7*	X					X			Only on Comparison Lists
17	*MYO15B*	X		X*	X					
6	*MYO6*			X		X*	X			
2	*NEB*				X		X			Only on Comparison Lists
2	*NMI*					X	X			Only on Comparison Lists
16	*NOD2*	X						X		Only on Comparison Lists
9	*NOL6*		X*						X*	
2	*OTOF*			X			X			Only on Comparison Lists
3	*PAK2*			X*				X		
5	*PCDH12*	X*						X*		
5	*PCDHB3*	X	X*							
20	*PCK1*		X*		X					
4	*PDHA2*						X	X*		
21	*PFKL*	X			X*					
19	*PLEKHG2*		X			X				Only on Comparison Lists
19	*PLIN4*			X*	X					
15	*POLG*			X			X			Only on Comparison Lists
13	*RBM26*	X		X*						
13	*SKA3*				X		X			Only on Comparison Lists
**6**	***SLC17A4***			X				X		Only on Comparison Lists
5	*SRA1*		X*		X					
17	*SREBF1*				X			X		Only on Comparison Lists
1	*SYCP1*			X	X*	X*		X		
6	*TBP*	X	X*		X			X*		
12	*TDG*	X						X		Only on Comparison Lists
12	*TMBIM4*			X*			X	X		
15	*TYRO3*				X		X			Only on Comparison Lists
1	*UBXN11*		X		X					Only on Comparison Lists
10	*UNC5B*		X				X			Only on Comparison Lists
7	*ZMIZ2*	X						X		Only on Comparison Lists
19	*ZNF226*		X			X*				
19	*ZNF738*				X*			X		
	Total	19(1)	11(6)	12(5)	18(5)	6(4)	17(0)	19(5)	2(2)	

Note. *on co-segregating variant list, total number of variants on co-segregating variant list in parentheses; ‘Only on Comparison List’ refers to variants not on the co-segregating variant list and not prioritized for confirmation, variants in bolded genes were prioritized because they are within the chr6 region previously linked to the TEGI (Rice, Smith, et al., 2009; Table S14a-S14e)

Table S9.

Summary of 44 Variants on 23 Genes Prioritized from Filtering Workflow 1 Cross-referenced List.

Chr	Unique Genes	# of var	rsID/ chromosomal location (hg38)	c.DNA	AA change	Variants in Family: Strict Lists	Variants in Family: Compare List
**Confirmed**							
1	*IQGAP3*	2	rs112144116	c.1684G>A	p.Ala562Thr	5886	
			rs147754283	c.1888C>T	p.Arg630Trp		4093
4	*PDHA2*	1	rs147966234	c.857G>C	pArg286Pro	5886	5463
5	*PCDHB3*	1	rs147754283	c.242C>T	p.Thr81Ile	4130	4093
6	*MYO6*	3	rs551348450	c.2743dupA	p.Gln918Thrfs*24	4379	
			chr6:75,857,098	c.1225G>A	p.Val409Ile	4132	
			rs573770611	c.271G>A	p.Ala91Thr		5463
9	*NOL6*	2	rs114465306	c.401C>T	p.Pro134Leu	4130	
			rs114110943	c.1096C>T	p.His366Tyr	5931	
15	*FURIN*	1	rs150925934	c.1390C>T	p.Arg462Trp	4075	5886
17	*BAHCC1*	2	rs200719992	c.7294C>G	p.Gln2463Glu	5931	
			rs369588790	c.732G>A	p.Arg2199Gln		4093
17	*MYO15B*	4	rs139077523	c.1615C>T	p.Pro539Ser	4132	
			chr17:75,625,226	c.8678A>C	p.Asn2893Thr		4093
			rs202034551	c.7726G>C	p.Ala2576Pro		4075
			chr17:75,619,800	c.7187+1G>A	splicing		4075
19	*ZNF226*	1	rs200990346	c.1036C>T	p.Arg346Cys	4379	4130
**Indel in everyone**							
1	*SYCP1*	1	chr1:114,994,979	c.2892dupA	p.Leu968Thrfs*5	4075, 4379	4132, 5886
6	*MYO6*	3	rs551348450	c.2743dupA	p.Gln918Thrfs*24	4379	
12	*TMBIM4*	1	chr12:66,138,156	c.520dupT	p.Tyr174Leufs*2	4132	5463, 5886
**Confirmed a different number of repeats in Sanger sequencing than called in exome data in most individuals**							
5	*PCDH12*	1	chr5:141,945,390	c.3545_3546 insCAGCAG	p.Ser1181_Arg1182 insSS	4093, 5886	
**NOT Confirmed**							
2	*HS6ST1*	2	rs199993343	c.T341G	p.Val114Gly	4130, 4075	
			chr2:128,318,170	c.C394G	p.Arg132Gly		4093
3	*PAK2*	1	rs201465227	c.G303C	p.Gln101His	4132	5886
13	*RBM26*	1	chr13:79,344,671	c.2100dupA	p.Q701Tfs*23	4132	4093
**Primers could Not be Optimized**							
8	*GPT*	2	chr8:144,506,908	c.C1262A	p.Pro421His	5886	
			chr8:144,506,631	c.1401-2A>C	splicing		4093
17	*MYO15B*	4	rs202034551	c.7726G>C	p.Ala2576Pro		4075
**No primers designed – due to reported protein expression**							
5	*SRA1*	2	rs148259347	Steroid receptor, associated with cancer growth (e.g., (Lin et al., 2017)		4130	
			rs35610885				4075
6	*TBP*	5	chr6:170561952-76	ALT = 0*		4130, 5886	4093, 4075
19	*BICRA*	4	chr19:47695363-65	ALT = 0*		4379	5463, 5886
19	*ZNF738*	1	chr19:21,383,393	ALT = 0*		4075	5886
19	*PLIN4*	2	chr19:4,512,672	Lipid droplets & misc. non-brain diseases (e.g. (Gasparin et al., 2018); additional references)		4132	
			chr19:4,511,759				4075
19	*PFKL*	2	rs61737076	Part of a cluster of intestine & liver – lipid metabolism genes^, SNP associated with hemoglobin A1c level (Barton et al., 2021; Lee et al., 2022)		4075	
			rs118106526				4093
20	*PCK1*	2	rs41302559	Part of a cluster of liver – oxidoreductase activity^, Gene associated with non-brain diseases (Beale et al., 2007; Xiang et al., 2022)		4130	
			rs367998997				4075

Note. AA = amino acid; light grey = not followed up in families, *0 called as alternate allele by GATK (Genome Analysis Toolkit), ^according to The Human Protein Atlas (proteinatlas.org)

Table S10.

Summary of Prioritized Variants from Filtering Workflow 1 Sequenced and Confirmed in Family Members.

Gene	rsID/ chromosomal location (hg38)	Family	TEGI Affected with variant	TEGI Affected Total in family	Confirmation Note
**Sequenced in Additional Probands**					
IQGAP3	rs112144116	5886	4	4	-from omnibus AFF dad
	rs147754283	4093	2	4	
PDHA2	rs147966234	5886	4	4	-from omnibus AFF dad
		5463	2	3	-not in either TEGI unaffected sibling
PCDHB3	rs147754283	4130	2	2	-from omnibus AFF mom
		4093	3	4	
NOL6	rs114465306	4130	2	2	-from omnibus AFF mom
	rs114110943	5931	2	2	-carried by 2 siblings–1 TEGI unaffected
BAHCC1	rs200719992	5931	2	2	-carried by 2 siblings–1 TEGI unaffected
	rs369588790	4093	3	4	
**Confirmed but not co-segregating and NOT selected for additional sequencing**					
MYO6	chr6:75,857,098	4132	1	6	
	rs573770611	5463	2	3	-from omnibus UNaff mom
FURIN	rs150925934	4075	3	4	-from omnibus UNaff mom
		5886	2	4	-in AFF twins, from omnibus AFF dad
MYO15B	rs139077523	4132	3	6	-branch 2 only
	chr17:75,625,226	4093	2	4	
	chr17:75,619,800	4075	2	4	
ZNF226	rs200990346	4379	1	3	-from omnibus UNaff dad
		4130	2	2	-NOT from omnibus AFF mom (dad NA)

Note. Confirmation notes include additional notes when relevant, such as when the variant is inherited from a parent affected on a standardized omnibus language measure or when a sibling who is unaffected on the TEGI carries or does not carry the variant; AFF = affected; UNaff = unaffected; NA = not available

Table S11a.

18 Rare Variants Prioritized in Family 4093 Shared by All Four Family Members Affected on the TEGI.

Gene	Chr	Genomic Position (hg38)	rsID	Notes
ARHGEF17	11	73360507	rs2298808	
ATP2A3	17	3937503	rs140980200	
FLT4	5	180621641	rs55667289	
GLYATL3	6	49512028	rs560399915	
MAGEC1	X	141906119	-	On ChrX
MAGEC1	X	141906127	-	On ChrX
MPDZ	9	141906119	-	
NFATC4	14	141906127	-	
PABPC1	8	100706893	rs79986761	Low quality genotypes
**PCDH12*	5	13136784	rs200891478	Same variant in 5886
PDE3A	12	24373757	rs142749204	
PLA2G3	22	100706893	rs79986761	
PRDX5	11	141945390	-	
RELN	7	20616336	rs141325069	
RSAD2	2	31140148	rs573436695	
TTC14	3	64319797	rs77269065	
VEPH1	3	103700962	rs149397714	
CCHC24	10	6887075	rs140690041	

Note. *variant prioritized for confirmation in the family members (located within one of the 23 genes of interest)

Table S11b.

31 Rare Variants Prioritized in Family 4130 Shared by Both Family Members Affected on the TEGI.

Gene	Chr	Genomic Position (hg38)	rsID	Notes
ADGRL2	1	81990423	rs72719419	
AIPL1	17	6425644	rs150427474	
C9orf85	9	71947067	rs142178034	
CDC7	1	91511686	-	
CORO2A	9	98157552	rs61741701	
DGKQ	4	967972	rs113007498	
DIO1	1	53894353	rs375309412	
DSPP	4	87615746-55	-	
DSPP	4	87615758-67	-	
FERMT3	11	64207494	rs149000560	
**HS6ST1*	2	128318223	rs199993343	Same variant in 4075, Diff. variant in 4093
IK	5	140658953	rs34433858	
METTL17	14	20992611	rs72661115	
MPO	17	58270865	rs35897051	
MPPED2	11	30411583	-	
**NOL6*	9	33470169	-	Different variant in 5931
PCDHB16	5	141183583	rs61742261	
**PCDHB3*	5	141100891	rs61739886	
PCF11	11	83168721	-	
*PCK1^*	20	57565383	rs41302559	Different variant in 4075
PECR	2	216081740	rs144581659	
RPL10	X	154400837	-	On ChrX
SCN1A	2	166045080	rs121918817	
SEMG2	20	45222191	rs138018319	
*SRA1^*	5	140552106	rs148259347	Different variant in 4075
SULT1C3	2	108259015	-	
*TBP^*	6	170561952	-	Different variants in 4093. 4075. 5886
TBX6	16	30088596	-	
UBE3A	15	25408686	-	
ZKSCAN7	3	44570999	rs373307729	
ZNF207	17	32360964	-	

Note. *variant prioritized for confirmation in the family members (located within one of the 23 genes of interest), ^variant located within one of the 23 genes of interest, but it was not ultimately prioritized for sequencing confirmation in the family members because it was likely a CNV or additional expression information excluded it

Table S11c.

31 Rare Variants Prioritized in Family 4132 Shared by Two or More Family Members from Different Branches Affected on the TEGI.

Gene	Chr	Genomic Position (hg38)	rsID	Notes
ABHD18#	4	128011984	-	
ART1	11	3660253	rs150574054	
ATN1	12	6936737	-	
BOD1L1	4	13614505	rs144761044	
C10orf67#	10	23239770	rs111911206	
DEPDC1	1	68496977	rs144782062	
G2E3	14	30605565	-	
GP1BA	17	4933948	-	
GRIN2B#	12	13611737	rs145021339	Candidate gene variant
KCNE2	21	34370500	rs2234916	
LRRN4	20	6041600	rs145844426	
MUC2#	11	1095060	-	False positive
**MYO15B#*	17	75589672	rs139077523	Different variant in 4093
NUMA1	11	72010801	-	
PABPC1	8	100709481	rs146200489	Confirmation was unsuccessful in previous projects
PABPC1	8	100709671	rs142985461	Confirmation was unsuccessful in previous projects
PABPC1	8	100709464	-	Confirmation was unsuccessful in previous projects
PADI2	1	17069250	rs139624393	
**PAK2*	3	196803031	rs201465227	Same variant in 5886
*PLIN4^*	19	4512672	-	Different variant in 4075
POLRMT	19	632849	rs139758373	
**RBM26*	13	79344671	-	Different variant in 4093
ROPN1L	5	10450114	-	
SLAIN1#	13	77746666	rs144139933	
SLC26A5	7	103421378	rs141952919	
SOX30	5	157626561	rs139465019	
SS18	18	26035040	rs147146752	
**TMBIM4*	12	66138156	-	Same variant in 5463, 5886
TTK	6	80007992	-	
TUBGCP3	13	112547552-57	-	
XDH	2	31366019	rs140007233	

Note. *variant prioritized for confirmation in the family members (located within one of the 23 genes of interest), ^variant located within one of the 23 genes of interest, but it was not ultimately prioritized for sequencing confirmation in the family members because it was likely a CNV or additional expression information excluded it, #variants were only called in two individuals affected on the TEGI, but genotypes were missing for affected members of the other branch, so the variant remained on the final prioritized list because the variants could be present in additional family members

Table S11d.

37 Rare Variants Prioritized in Family 4075 Shared by Three or More of the Four Family Members Affected on the TEGI.

Gene	Chr	Genomic Position (hg38)	rsID	Notes
BRD4	19	15273009	-	
CAPS	19	5915231	rs201449236	
CLEC4G	19	7730164	-	
CNTN5	11	100061213	rs201910584	
COL3A1	2	188997207	rs35795890	
DDI1	11	104036976	rs375454517	
DNAH1	3	52350578	rs61734644	
DNAH5	5	13844842	rs116524991	
DNHD1	11	6564423	rs552630821	
ELF2	4	139072021	rs17322140	
FAM149A	4	186167245	rs111681837	
**FURIN*	15	90880107	rs150925934	Same variant in 5886
GAS8	16	90031453	rs884928	
**HS6ST1*	2	128318223	rs199993343	Same variant in 4130, Diff. variant in 4093
HSD17B4	5	119531311	rs201560431	
ITIH2	10	7723566	rs76140242	
KDM1B	6	18197590	rs138145635	
MAP2K1	15	66436777	-	
MCM9	6	118913414	rs78231991	
MED4	13	48090420	-	
NDUFAF6	8	95035578	-	
NELL2	12	44665527	rs138454729	
NPHS1	19	35842196	rs143986233	
OLAH	10	15071877	rs141112464	
PCDHA3	5	140803137	rs149374718	
PCDHGB2	5	141361373	-	
*PFKL^*	21	44314011	rs61737076	Different variant in 4093
PRODH2	19	35806769	rs148996461	On ChrX
RBMX	X	136877987	-	On ChrX
RBMX	X	136877960	rs76812369	On ChrX
RPL10	X	154400837	-	
**SYCP1*	1	114994979	-	Same variant in 4132, 4379, 5463
TRIM56	7	101089563	rs148309415	
UNC13B	9	35231126	rs200386049	
UNC13B	9	35403557	rs201643678	
ZBTB25	14	64487702	rs142592421	
*ZNF738^*	19	21383393	-	Same variant in 5586

Note. *variant prioritized for confirmation in the families (located within one of the 23 genes of interest), ^variant located within one of the 23 genes of interest, but it was not ultimately prioritized for sequencing confirmation in the family members because it was likely a CNV or additional expression information excluded it

Table S11e.

17 Rare Variants Prioritized in Family 4379 Shared by Two or More of the Three Family Members Affected on the TEGI.

Gene	Chr	Genomic Position (hg38)	rsID	Notes
*BICRA^*	19	47695363-64	-	Different variant in 5463, 5586
*BICRA^*	19	47695363	-	Same variant in 5463, 5586
*BICRA^*	19	47695364	-	Same variant in 5463, 5586
*BICRA^*	19	47695365	-	Same variant in 5886
CD37	19	49336960	-	
CSMD2	1	33714622	-	
EFCAB13	17	47361523-26	-	
EFCAB13	17	47361521-22	-	
MTTP	4	99594765	-	
**MYO6*	6	75890140	rs551348450	Different variant in 4075, 5463
PABPC1	8	100709499	rs139094790	
RSPH6A	19	45795909	-	
**SYCP1*	1	114994979	-	Same variant in 4132, 4075, 5886
TMEM143	19	48342687	rs138056528	
UNC13A	19	17639084	rs200328448	
WFIKKN2	17	50839994	rs191998613	
**ZNF226*	19	44176298	rs200990346	Same variant in 4130

Note. *variant prioritized for confirmation in the families (located within one of the 23 genes of interest), ^variant located within one of the 23 genes of interest, but it was not ultimately prioritized for sequencing confirmation in the family members because it was likely a CNV or additional expression information excluded it

Table S11f.

6 Rare Variants of Large Effect Prioritized in Family 5463 Shared by All Three of the Four Family Members Affected on the TEGI.

Gene	Chr	Genomic Position (hg38)	rsID	Notes
ALDH7A1	5	126545018	rs61757684	
CCDC136	7	128801438	rs185493260	
MADCAM1	19	501762	-	
MADCAM1	19	501762	-	
PABPC1	8	100709671	rs142985461	Confirmation was unsuccessful in previous projects
PABPC1	8	128801438	rs185493260	Confirmation was unsuccessful in previous projects

Table S11g.

23 Rare Variants Prioritized in Family 5886 Shared by All Four of the Four Family Members Affected on the TEGI.

Gene	Chr	Genomic Position (hg38)	rsID	Notes
ADAMTS6	5	65470971	rs61736454	
BUD13	11	116762900	rs139478949	
CBR4	4	168990307	rs80133417	
FAM13A	4	89056962	-	
GCAT	22	37815274	rs150003624	
GNAS	20	58854572	-	
**GPT*	8	144506908	-	Different variant in 4093
**IQGAP3*	1	156551755	rs112144116	Different variant in 4093
ITGA10	1	145897563	rs116524970	
KDM6B	17	7847264	-	
MICU3	8	17105561	rs143132509	
NHLRC3	13	39047828	rs149175958	
NUP210L	1	153995093	rs199577888	

Gene	Chr	Genomic Position (hg38)	rsID	Notes
OPLAH	8	144058364	-	
**PCDH12*	5	141945390	-	Same variant in 4093
PDE9A	21	42765388	-	
**PDHA2*	4	95841007	rs147966234	Different variant in 5463
PTGFRN	1	116967102	rs201491047	
RPRD2	1	150471124	rs201498425	
SH3RF2	5	146049208	-	
STAB1	3	52520515	rs147953260	
TBP^	6	170561964	-	Same variant in 4130, 4075, Diff. variant(s) in 4130
UHRF1BP1L	12	100058491	-	

Note. *variant prioritized for confirmation in the families (located within one of the 23 genes of interest), ^variant located within one of the 23 genes of interest, but it was not ultimately prioritized for sequencing confirmation in the family members because it was likely a CNV or additional expression information excluded it

Table S11h.

18 Rare Variants Prioritized in Family 5931 Shared by Both Family Members Affected on the TEGI.

Gene	Chr	Genomic Position (hg38)	rsID	Notes
ARHGAP31	3	119414288	rs186621177	
ASPSCR1	17	82010826	rs202149445	
**BAHCC1*	17	81461957	rs200719992	Different variant in 4093
CCDC61	19	46008163	rs146892135	
COBLL1	2	164743733	-	
DHDH	19	48942538	rs140363616	
DTD2	14	31457378	rs17097904	
IFIH1	2	162278246	-	
IL15RA	10	5966288	rs149532559	
**NOL6*	9	33468803	rs114110943	Different variant in 4130
PABPC1	8	100709671	rs142985461	
PER2	2	238253082	-	
PFKP	10	3119938	rs41288721	
RBMX	X	136877987	-	
RPL10	X	154400837	-	
SDR42E1	16	81999895	rs201686460	
TKTL2	4	163472321	rs200478211	
TPM2	9	35685296	rs150120234	

Note. *variant prioritized for confirmation in the families (located within one of the 23 genes of interest), ^variant located within one of the 23 genes of interest, but it was not ultimately prioritized for sequencing confirmation in the family members because it was likely a CNV or additional expression information excluded it

Table S12.

Number of Candidate Gene Variants: Familywise Filtering Workflow 2b.

	**4093**	**4130**	**4132**	**4075**	**4379**	**5463**	**5886**	**5931**
Variants with gnomAD global MAF < 0.07, not synonymous and not segmentally duplicated	12,041	5,084	28,643	18,543	18,410	16,425	21,331	29,008
Variants on candidate genes in 1 individual affected on TEGI	67	26	136	68	62	55	91	36
Keep: exonic	16	12	46	24	13	17	24	18
shared by 2+ individuals with TEGI phenotype	6	8	15	4	2	3	11	1
Keep: CADD pred score ≥ 20	2	2	9	0	2	2	7	0
Keep: positive GERP score	2	2	8	0	2	2	7	0
Remove: bioinformatic prediction score sums[1] = 0/5, 1/5, 0/4, 1/4, 0/3	2	2	7	0	1	2	4	0
							Total variants	18
							Total unique variants	14
							Total unique genes	13

Note. MAF = minor allele frequency; GERP = Genomic Evolutionary Rate Profiling; CADD = Combined Annotation Dependent Depletion; [1]Prediction score sum includes = SIFT, PolyPhen-2, Mutation assessor, PROVEAN, and MutationTaster2

Table S13.

Summary of Confirmation Notes for the 13 Variants Prioritized from Filtering Workflow 2 Output Sequenced in Family Members.

Gene	rsID/ chromosomal location (hg38)	c.DNA	AA change	Family	TEGI Affected with variant	TEGI Affected Total in family	Confirmation Note	Previous Phenotype
**Confirmed and sequenced in additional probands**								
FLNB	rs116826041	c.6959T>C	p.Ile2319Thr	4132	3	6	-confirmed in branch 1	SLI/DLD
GLI3	rs35364414	c.4609C>T	p.Arg1537Cys	4093,<br>4130,<br>4132	3<br>3<br>3	4<br>2<br>6	-confirmed in branch 1 (not in TEGI UNaff sibling)	Aphasia
KMT2D	rs146044282	c.10256A>G	p.Asp3419Gly	5463	2	3	-from omnibus AFF dad	SLI/DLD
**Confirmed but not co-segregating and NOT selected for additional sequencing**								
SCN9A	rs202110802<br>rs200391162	c.317T>C<br>c.1109C>T	p.Leu106Ser<br>p.Thr370Met	4132<br>5886	2<br>3	6<br>4	-proband's child (TEGI AFF) did not inherit<br>-from omnibus UNaff dad	SLI/DLD
KIAA0319	rs113411083	c.2164G>A	p.Arg722Trp	5886	2	4	-not in the TEGI AFF twin siblings	DL, SSD, SLI/ DLD, CAS, ASD
ZFHX4	rs142555710	c.9341G>A	p.Gly3114Asp	4093	2	4		CAS
FAT3	rs80293525	c.2681G>A	p.Arg894Gln	4379	2	3	-from omnibus UNaff mom, in TEGI UNaff sibling	SLI/DLD
GRIN2B	rs145021339	c.1768G>A	p.Ala590Thr	4132	1	6	-not present in two TEGI AFF in which variant was called in WES output	ID, ASD
COL4A2	rs117412802	c.3368A>G	p.Glu1123Gly	5463	2	3	-from omnibus UNaff mom	SLI/DLD
NOP9	chr14:24300643	c.483_484 '-'>(GAG)3	p.Glu169_ Asp170 ins(Glu)3	4130,<br>4132			-(GAG)$_9$GA genotype in TEGI AFF<br>-(GAG)$_9$GA or (GAG)$_{10}$GA genotype in TEGI AFF	SLI/DLD
CHRNA3	chr15:78601954	c.688G>A	p.Asp230Asn	5886	3	4	-from omnibus UNaff mom	SLI/DLD
GRIN2A	chr16:9763667	c.3877G>T	p.Asp1293Tyr	4132	2	6	-proband and proband's child only	Focal epilepsy with speech disorder, with or without ID
**No primers designed**								
PTEN	chr10:87864103-4	ALT = 0*		4132,<br>5886				ASD

Note. AA= amino acid; light grey = not followed up in families;, *0 called as alternate allele by GATK (Genome Analysis Toolkit); #different number of repeats than called by exome data; SLI/DLD = specific language impairment/developmental language disorder; DL = dyslexia; CAS = childhood apraxia of speech; ID = intellectual disability; SSD = other speech sound disorders; ASD = autism spectrum disorder; grey = not relevant due to different number of repeats in different affected family members; Confirmation notes include additional notes when relevant, such as when the variant is inherited from a parent affected on a standardized omnibus language measure or when a sibling who is unaffected on the TEGI carries or does not carry the variant; AFF = affected; UNaff = unaffected; NA = not available

Table S14a.

Number of Variants on Chromosome 3q13.12-q13.31: Familywise Filtering Workflow 2a.

	**4093**	**4130**	**4132**	**4075**	**4379**	**5463**	**5886**	**5931**
chr3:107271010-116470713	55	11	55	48	44	38	51	22
Remove: segmentally duplicated	55	11	55	48	42	38	51	22
Keep: gnomAD global MAF < 0.07	48	8	47	40	36	35	45	21
Remove: synonymous	46	7	44	36	33	32	44	15
Keep: carried by ≥ 2 family members affected on TEGI	12	4	13	11	5	11	10	4
Keep: exonic OR splicing	3	3	2	2	1	4	5	4
Keep: CADD pred score ≥ 20	3	1	1	1	1	1	3	2
Keep: positive GERP score	3	1	1	1	1	1	3	1
Remove: bioinformatic prediction score sums[1] = 0/5, 1/5, 0/4, 1/4, 0/3	3	0	1	1	1	1	3	0

Total number of shared genes = 3
*1 variant = genotype quality is very low, and many genotypes were called
*1 variant is carried by an unaffected individual in family 5463
(listed in full in Table A 13)

Note. MAF = minor allele frequency; GERP = Genomic Evolutionary Rate Profiling; CADD = Combined Annotation Dependent Depletion; [1]Prediction score sum includes = SIFT, PolyPhen-2, Mutation assessor, PROVEAN, MutationTaster2

Table S14b.

Variants prioritized familywise within RD region: chr3q13.12-q13.31, previously significantly associated with TEGI phenotype.

**Family(s)**	**Gene**	**Genomic Position (hg38) chr3**	**rsID**
4093	*CCDC191*	114046612	rs138852073
4093, 5886	*USF3**	113658634	-
4075	*GTPBP8*	112991286	rs114429530
5463, 5886	*CIP2A*	108585142	rs34788499
489	*SPICE1*	113468801	rs73239152

Note. *cross-referencing step revealed that four individuals with low TEGI performance carry a variant on the same gene

Table S14c.

Number of Variants on Chromosome 6p21.1-p22.3: Family Filtering Workflow 2a.

	**4093**	**4130**	**4132**	**4075**	**4379**	**5463**	**5886**	**5931**
chr6:21725992-41417847	121	29	207	120	86	117	128	79
Remove: segmentally duplicated	111	25	194	113	78	110	120	75
Keep: gnomAD global MAF < 0.07	98	18	173	95	70	88	104	70
Remove: synonymous	81	16	151	86	66	80	96	46
Keep: carried by ≥ 2 family members affected on TEGI	14	4	16	13	6	16	22	0
Keep: exonic OR splicing	3	4	6	5	0	1	7	0
Keep: CADD pred score ≥ 20	3	3	4	3	0	0	4	0
Keep: positive GERP score	3	3	4	2	0	0	4	0
Remove: bioinformatic prediction score sums[1] = 0/5, 1/5, 0/4, 1/4, 0/3	2	2	3	2	0	0	3	0
	Total number of shared genes = 2 (3 unique variants) (listed in full in Table A 14)							

Note. MAF = minor allele frequency; GERP = Genomic Evolutionary Rate Profiling; CADD = Combined Annotation Dependent Depletion; [1]Prediction score sum includes = SIFT, PolyPhen-2, Mutation assessor, PROVEAN, MutationTaster2

Table S14d.

Variants prioritized familywise within RD region: chr6p21.1-p22.33q13.12-q13.31, previously significantly associated with TEGI phenotype.

**Family(s)**	**Gene**	**Genomic Position (hg38) chr6**	**rsID**	**Note**
4093	*HIST1H4I*	27139442	-	
4093	*BTN2A1*	26458677	-	
4130	*DNAH8*	38938181	rs61757218	
4130	*TSPO2*	41042779	rs41273356	
4132	*HFE*	26092913	rs1800562	
4132, 4075	*FGD2**	37011707	-	
4132	*SLC17A4**	25776649	rs376955338	
5886		25773649	rs145440760	
4075	*DAAM2*	39878255	-	
5886	*KCNK16*	39317794	rs146487869	
5886	*KIAA0319*	24566725	rs113411083	Candidate gene list

Note. *cross-referencing step revealed that four individuals with low TEGI performance carry a variant on the same gene

Table S14e.

Primers used for confirmation of variants in RD regions via sequencing.

Family	Prioritization Workflow	Gene	Chr	Variant Location (hg38)	Forward Primer	Reverse Primer	PCR product size	Optimized Tm	Direction for seq
4093, 5886	RD regions	*USF3*	3	113658634	CAAGTACTGACTGTGTTTCTGAGG	GGAATGATGCTGTGGAGGAT	296	56°	Fwd
4132, 4075		*FGD2*	6	37011707	aacctcccctgccttctttt	cctaggaggctgaacctagc	400	56°	Fwd
4132		*SLC17A4*	6	25776649	cagtctgtccaaggttgtctg	ggggtaggaggacaaacaca	291	56°	Fwd
5886				25773649	tcttccagGACTGTTCACCA	aggtcctgcctggtcctaat	291	56°	Fwd

Note. *primer pair used to initially amplify genomic region, ^primer pair used internally to amplify prioritized variant
